# The Potential of Plant Phenolics in Prevention and Therapy of Skin Disorders

**DOI:** 10.3390/ijms17020160

**Published:** 2016-02-18

**Authors:** Magdalena Działo, Justyna Mierziak, Urszula Korzun, Marta Preisner, Jan Szopa, Anna Kulma

**Affiliations:** 1 Faculty of Biotechnology, University of Wroclaw, Przybyszewskiego 63/77, 51-148 Wroclaw, Poland; magdalena.dzialo@uwr.edu.pl (M.D.); juststw@o2.pl (J.M.); urszula.korzun@uwr.edu.pl (U.K.); marta.preisner@gmail.com (M.P.); szopa@ibmb.uni.wroc.pl (J.S.); 2 Department of Genetics, Plant Breeding and Seed Production, Faculty of Life Sciences and Technology, Wroclaw University of Environmental and Plant Sciences, Plac Grunwaldzki 24A, 53-363 Wroclaw, Poland

**Keywords:** phenolic compounds, anti-aging properties, skin diseases, wound healing, antioxidant, anti-inflammatory, antimicrobial, anti-carcinogenic

## Abstract

Phenolic compounds constitute a group of secondary metabolites which have important functions in plants. Besides the beneficial effects on the plant host, phenolic metabolites (polyphenols) exhibit a series of biological properties that influence the human in a health-promoting manner. Evidence suggests that people can benefit from plant phenolics obtained either by the diet or through skin application, because they can alleviate symptoms and inhibit the development of various skin disorders. Due to their natural origin and low toxicity, phenolic compounds are a promising tool in eliminating the causes and effects of skin aging, skin diseases, and skin damage, including wounds and burns. Polyphenols also act protectively and help prevent or attenuate the progression of certain skin disorders, both embarrassing minor problems (e.g., wrinkles, acne) or serious, potentially life-threatening diseases such as cancer. This paper reviews the latest reports on the potential therapy of skin disorders through treatment with phenolic compounds, considering mostly a single specific compound or a combination of compounds in a plant extract.

## 1. Introduction

The skin has many functions in humans, especially in the defense against physical, chemical or biological factors. The skin takes part in regulation of water and electrolyte homeostasis and plays a secretory role. Moreover, it is involved in perception and immunological response of the whole organism [1]. Disorders or damage of such an extensive and multifunction organ as skin can be a serious threat to patient health or even life.

The skin of the average adult occupies a surface area of approximately 1.5–2.0 m^2^ and constitutes one tenth of human body mass [2]. It is made from different layers separated by basal membrane, under which is located the subcutaneous tissue (hypodermis) consisting of fat and connective tissue. Other skin components are sweat and sebaceous glands, hair follicles and nails [1]. Epidermis is formed by several types of cells, among which keratinocytes are predominant. The other epidermal cells are melanocytes, Merkel cells and Langerhans cells [1,2]. The dermis is made of collagen fibers and elastin, fibronectin, proteoglycans, and glycoaminoglycans. However, the main component of this layer is fibroblasts [1,2,3].

Recently, many reports on the potential effectiveness of phenolic compounds in the prevention or attenuation of skin disorder symptoms and reduction of the healing time have been published [4,5,6,7]. Phenolic compounds are found to be one of the most important groups of plant secondary metabolites, due to their great participation in morphological development, physiological processes and reproduction. These phytochemicals are synthesized through the pentose phosphate, shikimate and phenylpropanoid pathways. Phenolics are mostly known for their broad spectrum of biological properties, which are due to their molecular structure. The main core of phenolic compounds is formed by at least one phenol ring, in which the hydrogen is usually replaced by a more active residue, such as hydroxyl, methyl or acetyl. The variable biological properties of the phenolics result from the pattern and the degree of the substitutes. Usually in plants these compounds contain more phenolic rings, and thus they are called polyphenols [8,9,10,11].

**Figure 1:**
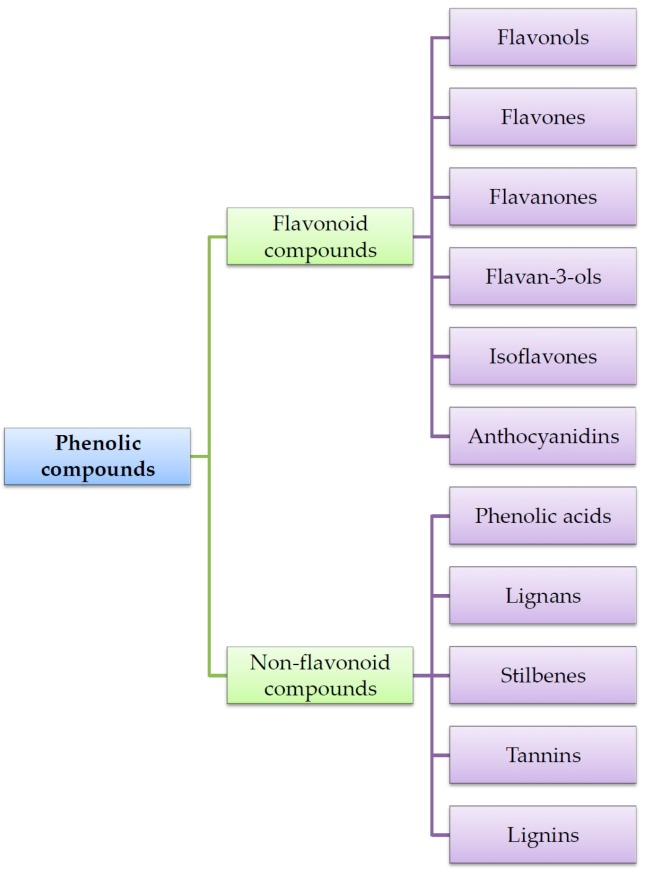
The classification of phenolic compounds.

Currently, approximately 8000 different structures of plant phenolics are known. The most common classification of phenolic metabolites distinguishes the flavonoid and non-flavonoid compounds. The chemical structure of flavonoid compounds is based on two aromatic rings connected by a bridge consisting of three carbons (C_6_-C_3_-C_6_). Flavonoids are divided into six main subclasses: flavonols, flavones, flavanones, flavan-3-ols, isoflavones and anthocyanidins. In the physiological state the flavonoids occur usually in association with sugar as glycosides. The second class of plant phenolics—non-flavonoid metabolites—consists of the following subgroups: phenolic acids (hydroxybenzoates C_6_-C_1_, hydroxycinnamates C_6_-C_3_), lignans (C_6_-C_3_)_2_ and stilbenes C_6_-C_2_-C_6_ [8,11]. Two other subclasses of non-flavonoids are tannins and lignins. These compounds occur mainly as complicated biopolymers; hence they lack a defined primary carbon base, and the chemical structure is unique for the particular polyphenol [12,13]. The classification of phenolic compunds is presented in Figure 1.

Flavonoids comprise the largest and most diverse group of phenolic compounds in plants. In the plant cell, flavonoids occur most frequently as glycosides dissolved in the vacuolar juice (mainly in the *O*-glycoside form, rather than *C*-glycosides). They can also crystallize around the epidermis. In many plant species anthocyanins accumulate in the vesicles developed in the vacuole. In the dicotyledonous plants, flavonoids are very often the only metabolites which show pharmacological activity [14]. The main sources of flavonoids in the diet are fruits and vegetables. They occur also in certain grains, seeds, and spices, as well as in wine, tea, coffee, cocoa, and herbal essences [15,16].

The most common phenolic acids in plant tissues are hydroxycinnamic acids. This broad class includes caffeic acid, chlorogenic acid, *o*-, *m*- and *p*-coumaric acids, ferulic acids, and sinapic acids [17,18]. In plants the mentioned acids occur mostly in the associated form as esters or glycosides, for example as the lignin component. Particular hydroxycinnamic acids occur in the ester form associated with carboxyl acids or glucose [15,19,20,21,22]. One of the most widespread hydroxycinnamic acids is caffeic acid, which occurs in coffee, apples, potatoes, spinach lettuce, cabbage, olive oil, wine and tobacco leaves [23,24,25,26]. The second important group of phenolic acids is hydroxybenzoic acids, such as gallic acid, p-hydroxybenzoic acid, protocatechuic acid, vanillic and syringic acids [18,27,28,29]. The plant hydroxybenzoic acids occur mostly in the glycoside form. In plant tissues phenolic acids can be bound to various compounds, e.g., flavonoids, fatty acids, sterols and cell wall polymers [28].

The other phenolic compounds widely distributed in plants are tannins, which may occur as hydrolyzable tannins (formed in the pathway of the phenolic acids with sugar polymerization) and condensed tannins (combination of flavonoids) [30,31,32,33]. The main lignan compounds are secoisolariciresinol, lariciresinol, pinoresinol and matairesinol. These compounds are phenylpropanoid dimers. They mostly occur in the seed of flax and sunflower [34,35,36,37], but also small amounts of them can be found in grains, vegetables, fruits, nuts, tea and coffee [38,39,40,41]. Resveratrol is a representative compound of another group of phenolics—stilbenes [42,43,44]. Resveratrol (*trans*-3,5,4’-trihydroxystilbene) is present in many plant species, including those that are popular components of the human diet such as grapes, peanuts, and berries. Plants synthetize it as a part of defense mechanisms against mechanical injury, pathogen infection, and UV radiation. The structure of resveratrol, which consists of two aromatic rings, allows for two orientations, *trans*- and *cis*-isomers, thanks to the existence of a styrene double bond connecting the two rings. Being the preferred steric form, the *trans*-isomer of resveratrol is known for its better biological activity when protected from high pH and UV radiation [45,46]. A study on the connection between moderate red wine consumption and a low incidence of cardiovascular disease was reported. The scientist, who called the observed phenomenon the “French Paradox”, speculates that resveratrol is most probably responsible for this. Indeed, it was found out that resveratrol is of great importance in the treatment of cardiovascular diseases and cancers, as well as degenerative disorders [46,47]. Table 1 presents examples of particular plant phenolic compounds occurring in the human diet.

**Table 1:** The occurrence of phenolic compounds in predominantly edible plants and plant-derived products.

Phenolic Compounds		Occurrence in Plants	References
Flavonoids	Flavonols	Apples, oranges, grapefruits, black grapes, black elderberries, blueberries, cranberries, cabbage, lettuce, broccoli, radish, chives, onion paprica, chicory, green tea, red wine, *Ginkgo biloba* leaves, *Morus alba* leaves	[15,16,48,49,50,51,52,53]
	Flavones	Selery, cayenne pepper, red paprica, parsley, thyme, lemon, rose hip, peppermint	[15,16,51,52,53,54]
	Flavanones	Tomatoes, mint, nigella seeds, citrus fruits (mainly oranges and grapefruits)	[15,16,51,52,53,54]
	Flavanols	Tea, red wine, chocolate, apples, kiwi	[15,16,51,52,53]
	Isoflavones	Soy, soy products, legumes	[15,16,51,52,53,55,56,57,58]
	Antocyjanidins	Cherries, strawberries, grapes, red wine, black currant, black elderberries, chokeberries, blueberries, red cabbage, rhubarb, radish, red onion	[15,16,51,52,53,59]
Phenolic acids	Hydroxycinnamic acids	Apples, pears, plums, cherries, apricots, peachs, black currant, blueberries, *Ginkgo biloba* and *Morus alba* leaves, tobacco leaves, potatoes, spinach, lettuce, cabbage, bean, radish, potatoes, broccoli, curly kale, asparagus, olive oil, wine, coffee, citrus juice, grains	[20,23,30,60,61,62,63,64,65,66,67,68,69,70]
	Hydroxybenzoic acids	Grapes, black currant, blackberries, lingon berries, strawberries, raspberries, onion, tea	[20,69,70,71,72]
Tannins		Green and black tea, red wine	[32,33,48,73,74]
Stilbens		Grapes, mulberries, peanuts, berries	[42,43,44,56,75,76,77,78,79,80]
Lignans		Flaxseed, sunflower seeds, sezame seeds, grains, carrot, onion, chives, apples, cherries, blueberries, strawberries, nuts, tea, coffee	[34,35,38,39,43,81,82,83,84]

**Figure 2:**
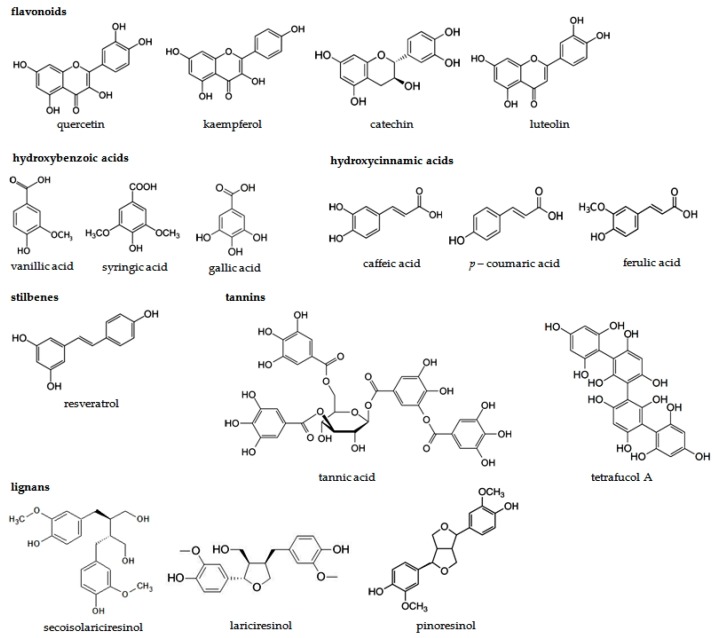
The examples of molecular structures of the most common phenolic compounds.

Primarily the chemical structure of plant phenolics determines their physicochemical properties. The examples of molecular structures of the most popular phenolic compounds are presented in the Figure 2. Phenols are similar to alcohols, but due to the aromatic ring they form stronger intermolecular hydrogen bonds, which enhance their water solubility and raise their melting and boiling points [85]. The plant phenolic compounds present a variety of colors from colorless to intense vibrant dyes such as red or violet. In the plant cells phenolics are stored near the chloroplast or they are accumulated in vacuoles, where they may polymerize and strengthen the cell wall [86].

The dynamics of compounds’ permeability through the skin depends on both biological factors (age, skin condition, cardiovascular functions and metabolism) and physicochemical ones, such as the partition coefficient between the stratum corneum and the vehicle and the related lipophilicity, as well as size, spatial structure of the molecule, polarity and load [87,88]. The phenolic compound’s permeability depends on the subclass of phenolics to which the particular compound belongs, the molecular size, structure and whether it occurs in the glycoside or aglycone form, as well as the other components of the formulation in which it is delivered [14,88,89,90]. Following the epidermis penetration, polyphenolic compounds may undergo non-enzymatic or enzymatic reactions [91,92].

The penetration of the active molecules may occur in the several ways: transepidermal, through the cells, intercellularly, transfollicular, through the sweat glands and hair follicles. Transcellular penetration takes place along the connections between corneocytes and is possible only for small molecules soluble in both water and fats. The lipophilic and amphiphatic compounds penetrate the skin through the intercellular way consisting of lipids. The penetration through the skin appendages is less important, because of the secretions flow, which direction is opposite to the direction of movement of the active substance. However, due to the fact that the skin acts as a barrier, most of the active ingredients delivered topically on the skin have a low natural permeability. In order to increase the permeability of the active compounds a number of physical methods (*i.e.*, electromechanical, electroporation, iontophoresis, or occlusive dressings) and chemical methods (including, the addition of chemical compounds that interact with components of the skin that cause a reversible modification of disrupting the barrier function and resulting in an increase in its permeability) were developed [93,94].

Such properties as improved topical absorption, performance and sensory factors of phenolic compounds products can be improved with the application of the newly developed encapsulation approaches, e.g., liposomes, phytosomes, transferosomes, nanoemulsions, nanoparticles, microemulsions, nanocrystals, and cubosomes. Textiles used in cosmetic and pharmaceutical applications might be a carrier of phenolic compounds and can act as the means of delivering the active substance directly to the irritated skin [95].

The purpose of this review is to present data on health-promoting properties of plant phenolics, both isolated pure components and mixtures naturally occurring in plant extracts, in the context of prevention and treatment of skin disorders, such as the premature effects of aging, skin diseases, as well as severe skin damage in the form of injuries. The collected data are taken from recent reports of the use of skin-protective natural phenolic compounds, not only provided in the diet, but also as components of cosmetic formulations.

## 2. Health-Promoting Activity of Phenolic Compounds Based on Antioxidant, Anti-Inflammatory and Antimicrobial Mechanisms

As is widely known, plant secondary metabolites such as phenolic compounds, besides the beneficial impact on the plant host, can be effective for humans in treating a variety of disorders. The most common properties of polyphenols—antioxidant, anti-inflammatory and antimicrobial—indicate that they deserve recognition in natural medicine and may be highly effective in treatment of various skin problems. Those three mentioned properties constitute the main potential mechanisms of action against various skin disorders.

### 2.1. Antioxidant Activity

**Figure 3:**
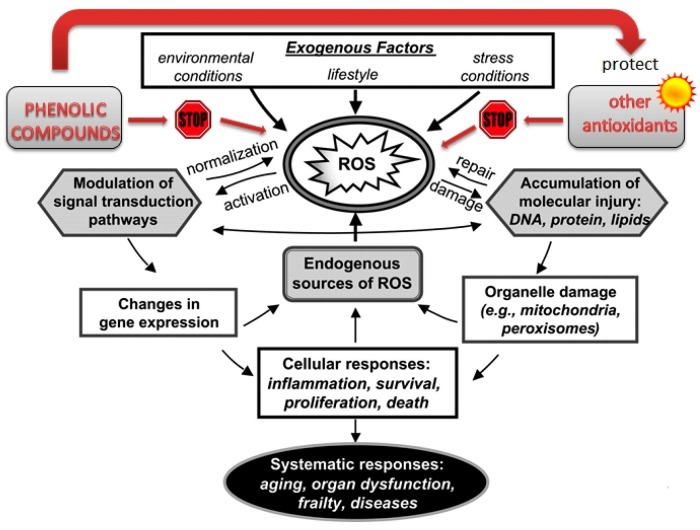
The scheme of factors involved in the formation of free radicals and a cellular response to reactive oxygen species (ROS). The red arrow and the text in red emphasize the importance of phenolic compounds, other antioxidants and the relationship between them. The sun signifies protection of other antioxidants by phenolic compounds.

It has been known for a long time that phenolic compounds occurring naturally in plants present a broad spectrum of health-promoting properties resulting from their biological activity. Certainly, these properties include antioxidant activity. There is no doubt that oxygen is an extremely important element for every living organism. However, reactive oxygen species (ROS) may be toxic and mutagenic. The reactive oxygen forms include superoxide, singlet oxygen, hydrogen peroxide and hydroxyl radical. Figure 3 presents the factors and mechanisms of oxidative stress. Excessive production of ROS can lead to oxidative stress triggering damage in cell structures, including lipids, proteins and DNA. This damage may cause many disorders such as cancer, inflammation, cataract, hypertension, diabetes, cardiovascular disease, Parkinson and Alzheimer diseases [96,97]. Reactive oxygen species can also negatively influence some immunological processes and aging, as well as pathophysiological mechanisms leading to skin inflammatory disorders [98]. Oxidative stress is understood as the disturbance of the homeostasis between reactive oxygen forms and the antioxidative defense system in the organism [99].

Antioxidant activity of phenolic compounds is associated with the annular structure of the molecule, conjugated double bonds and the presence of functional groups in the ring. The antioxidant activity of phenolics is possible through various mechanisms of action: inhibition of the ROS formation and the ROS trapping and the extinction of singlet oxygen; and reducing the chelated metal ions (which are the catalysts for reactions leading to the formation of ROS), interrupting the cascade of free radical reactions in lipid peroxidation and protecting the other compounds with antioxidant activity [100,101,102,103].

Skin is well equipped with two crucial means of defense against oxidative stress: antioxidant enzymes (catalase, glutathione peroxidase and peroxide dismutase) and non-enzymatic molecules (vitamins, ubiquinone, glutathione) [104]. However, often the endogenous defense system against ROS is insufficient. Thus it is recommended to increase the amount of natural antioxidants through the diet or external application. As an example of natural exogenous antioxidants we can give antioxidant vitamins (especially vitamins C and E), lipoic acid, coenzyme Q, melatonin, resveratrol, curcumin and other polyphenols [105]. These compounds are safe and more biologically active than synthetic antioxidants [98].

### 2.2. Anti-Inflammatory Properties

Every day, our organism is exposed to external factors which may cause various types of damage, irritation or allergies. The body's defensive reaction against the negative effects of these factors is inflammation. During the complex process of inflammation an excess of free radicals is produced. The formation of reactive oxygen and nitrogen species is associated with the triggering of biological responses to activation of the transcription factor AP-1 and nuclear transcription factor kappa B (NF-κB) [106]. These factors regulate secretion of signaling molecules, such as pro-inflammatory cytokines and interleukins, which lead to skin inflammation, appearing as redness and swelling of the inflamed area. The crucial functions of polyphenols are inhibition of pro-inflammatory mediators, neutralization of free radicals, ROS, RNS, and thus inhibition of lipid peroxidation [107].

During inflammation, arachidonic acid is released from the cell membrane phospholipids. The enzyme involved in this reaction is phospholipase A2 (PLA2), which is stimulated by oxidative stress. The released arachidonic acid is transformed by either the cyclooxygenase or lipoxygenase pathway. Polyphenols may inhibit both reactions, mostly due to the interruption of substrate binding to the enzyme by disruption of the hydrogen bonding system or due to chelation ions in the active center of the enzyme [108].

### 2.3. Antimicrobial Action

Phenolic compounds possess potent antifungal, antiviral and antibacterial activity [109]. Many types of infections or diseases, including the dermal kind, are treated with a broad activity spectrum antibiotic. It may lead to the negative influence of antibiotics on natural microflora of the skin and lead to resistance of many bacterial strains [110]. Thus the activity of polyphenols has special significance in the case of strains resistant to antibiotics, e.g., *Staphylococcus aureus* resistant to methicillin, enterococci resistant to glycopeptide antibiotics and vancomycin, pneumococci resistant to β-lactam and macrolides, and *Pseudomonas aeruginosa* with its defense mechanism against phagocytic activity of polymorphonuclear leucocytes [109,111,112]. Bacteria of the genera *Staphylococcus*, *Pseudomonas* and *Enterococcus* are the most frequent causes of hospital infections of skin wounds such as ulcers, bedsores or burns that result in many healing problems [113,114]. Recently over 90% of staphylococci, pneumococci and enterococci isolated from serious infections were found to be resistant to antibiotics; thus the demand for antibacterial products is still rising. These products may be used for multistrain bacterial infections, without causing a simultaneous toxic effect on human tissues [109]. The antibacterial properties of phenolics may result from the mechanism of their action on cell membranes [115]. Recently, it was reported that methanol extracts from *C. mucronatum* leaves have antibacterial properties against *Streptococcus pyogenes*, *Staphylococcus aureus*, *Pseudomonas aeruginosa*, *Bacillus cereus* and *Bacillus subtilis*. Regarding the phytochemical composition of *C. mucronatum* leaves, the presence of vitexin, isovitexin and tannins was confirmed. It was also shown that an ethanol extract from these leaves could potentially be used to treat wounds [116].

## 3. Anti-Aging Properties of Plant Phenolics

Changes in the skin are the most visible signs of aging. Loss of skin elasticity is manifested mostly as wrinkled, dry and flaccid skin. The appearance of the epidermis is affected by hyperpigmentation or inflammation (age spots). The skin condition is frequently the result of many aspects, such as genetics, environmental factors, nutrition, alcohol abuse or smoking. The main mechanism of skin cell disturbance is based on oxidative stress reactions [117].

Nowadays, people endeavor to slow down the aging process by proper diet, doing sports and using cosmetics. As already mentioned, phenolic compounds possess a broad spectrum of biological activities. Dietary phenolics may influence the internal organs, as well as the human skin [118,119]. However, the most effective way of skin treatment is surface and topical application (cosmeceuticals) [120].

**Figure 4:**
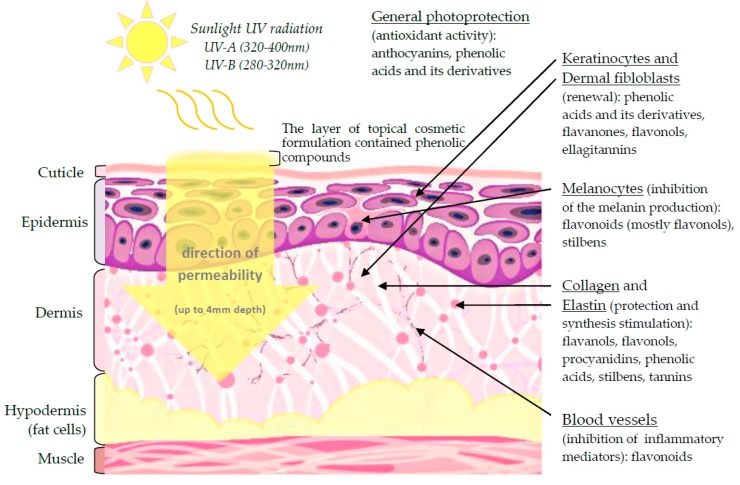
The scheme presents the cross-section of the skin structure and the specific influence phenolic compounds (delivered in the cosmetic formulation form) on dermal tissue components. Black arrows indicate the particular component of skin tissue, according to which are listed the main classes of phenolic, effective in prevention or treatment signs of skin-aging. The yellow arrow indicates the direction and the depth of the cosmetic formulation permeability through the skin. The various layers of the skin structure are indicated on the left. The scheme is based on data described in the review.

Since consumers prefer to use more natural compounds on their skin, plant phenolic compounds are a promising target for new dermal cosmetics that possess the ability to maintain the skin homogeneity and a proper, healthy look due to effective skin cell renewal, elastin and collagen stimulation and inhibition of excessive melanin synthesis (Figure 4). Below, we present examples of plant phenolic compounds as effective anti-aging agents.

### 3.1. Skin Cell Renewal

The epidermal skin cells possess the activity of continuous self-renewal and replacement of the dead cells. It was estimated that the human epidermis turnover time averages from 40 to 56 days. Unfortunately, the capacity for cell renewal rapidly decline with age [121]. However, this process can be slowed or even reversed by some biologically active agents, such as phenolic compounds.

It was reported that the phenolics present in *Populus nigra* buds showed favorable activity in the model of normal human dermal fibroblasts (NHDF). MS and NMR analyses revealed the presence of the following compounds in poplar bud extracts: six phenolic acids (caffeic, p-coumaric, isoferulic, di-*O*-methylcaffeic, cinnamic), three flavonoids (pinocembrin, pinobanksin and its derivative) and salicin. As expected, the identified compounds presented potent antioxidant activity. In order to evaluate the potential anti-aging activity of poplar buds extract, its transcriptional effects on genes involved in oxidative stress protection (catalase), cell renewal (Kruppel-like factor 10 (KLF10), E2F-4 transcription factor (E2F4), EGF response factor 1 (ZFP36L1)) and inflammatory response processes (chemokine (C-C motif) ligand 5 (CCL5)) were evaluated (Table 2). The expression levels were compared to the control (untreated aged fibroblasts, marked as 100%) and normal untreated fibroblasts. Among the five genes analyzed, only the gene linked to the inflammatory processes showed a decrease, which is consistent with stimulating and protective effect of *Poplar* phenolics. More importantly, three skin renewal genes involved in proliferation, differentiation, survival and DNA synthesis (known to be down-regulated in the normal NHDF cells) were increased almost 2-fold; thus they presented potent modulation of gene transcription [104]. The table 2 presents the levels of skin-related genes expression in NHDFs after the treatment with the studied extracts.

**Table 2:** The regulation of the skin-related genes expression in the normal human dermal fibroblasts (NHDF) cells treated with the extracts from black poplar buds, oak wood, mate leaves and benjoin resin. The results were referred to the control (untreated aged fibroblast cells, marked as 100%) [104,122].

Analyzed Plant Material (Phenolic Compounds)	Expression of Skin-Related Genes up-(+) or down-(−) Regulation (%)					References
	cat ^a^	KLF10 ^b^	E2F4 ^c^	ZFP36L1 ^d^	CCL5 ^e^	
Poplar bud (phenolic acids, flavonoids, salicin)	+130	+86	+151	+103	−39	[104]
Oak wood (ellagitannins)	+147	+81	+43	+101	−56	[122]
Mate leaf (caffeoyl derivatives)	+228	+83	+44	+70	−46	
Benjoin resin (phenolic acids)	+226	+48	+104	+69	−48	

^a^ catalase (antioxidative protection); ^b^ Krupper-like factor 10 (cell renewal); ^c^ E2F-4 transcription factor (cell renewal); ^d^ EGF response factor 1 (cell renewal); ^e^ chemokine (C-C motif) ligand 5 (inflammatory response).

Similar experiments on skin aging-related genes’ transcriptional modulation were performed in NHDF cells treated with phenolics from other plants, including oak wood, mate leaf and a plant-derivative product—benjoin resin. In the extracts, ellagitannins from oak wood, caffeoyl derivatives from mate leaf and phenolic acids from benjoin resin were identified. The expression tendency of evaluated genes was similar to the treatment with poplar bud extract (Table 2). A strong silencing effect was observed in the proinflammatory CCL5 gene. After treatment with extracts from oak wood, mate leaves and benjoin resin, the CCL5 gene expression was repressed by 56%, 46% and 48%, respectively. According to the proliferation marker genes (KLF10, E2F4 and ZFP36L1), all three genes showed high modulation after the treatment with phenolic compounds. For the oak wood extract, a potent increase was observed for KLF10 and ZFP36L1 gene expression. The expression of the KLF10 and E2F4 was similar between oak wood and mate leaf extracts treatment. Additionally, the expression of the E2F4 was higher after the benjoin resin treatment, in the comparison to oak wood and mate leaf phenolics action. Significant up-regulation was also noted for analyzed extracts in the antioxidant catalase gene [122].

### 3.2. Stimulation of Collagen and Elastin Synthesis

**Figure 5:**
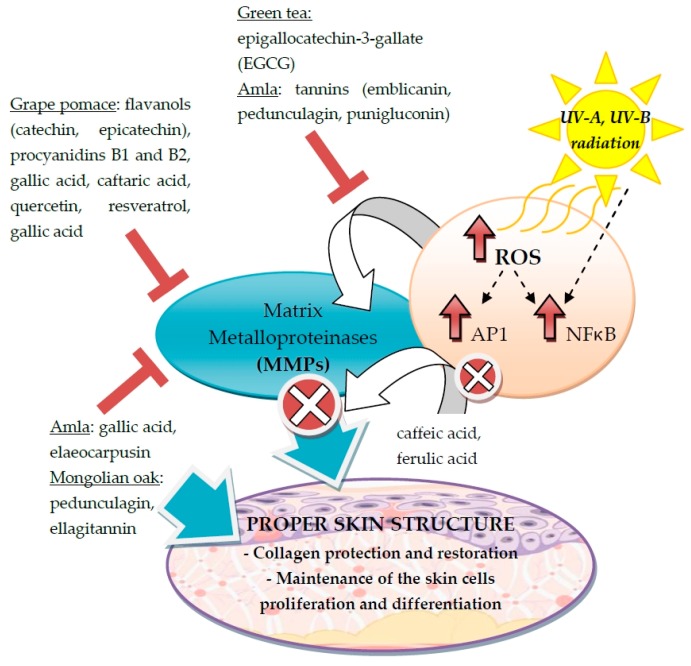
The factors and phenolic compounds involved in the maintenance of the proper skin structure through the regulation of matrix metalloproteinases (MMPs). The white cross in the red circle and the red signs in the letter “T” shape indicate the inhibition of the MMPs activity.

During aging, the extracellular matrix proteins are susceptible to excessive activity of proteolytic enzymes—matrix metalloproteinases (MMPs). This event concerns mostly collagen and elastin. Under proper physiological conditions the enzymes are regulated at the transcriptional level and by protein inhibitors. The imbalance in homeostasis leads to loss of integrity of the skin tissue, which may result in formation of wrinkles. It was reported that many polyphenols from plants (e.g., cocoa) exhibit inhibitory activity against collagenases and elastases, thus facilitating maintenance of proper skin structure [5]. Figure 5 presents the phenolic compounds involved in the preservation of the proper skin structure through the regulation of MMPs.

According to the research of Wittenauer *et al.*, the phenolic compounds identified in crude grape pomace from white wine might be useful in cosmetic formulations, due to their inhibitory properties against matrix metalloproteinases [4]. The compounds determined in the extract belong predominantly to the subclasses of flavanols catechin, epicatechin and procyanidins B1 and B2. Minor metabolites, but crucial in inhibition, were gallic and caftaric acids, quercetin glycosides and stilbene *trans*-resveratrol. The strongest activity was noted for gallic acid found in the first fraction of polar free phenolic acids. The second fraction containing procyanidins and catechins also revealed inhibitory action against proteolytic enzymes, especially collagenase. Despite effective suppressive activity against MMPs, the use of some polyphenols might be limited due to their high molecular weight and lower permeability potential through the skin layer [4].

Also important in protection of extracellular proteins is synthesis of enzymes in the inactive form (proenzymes) that require specific cleavage to release the active protease. An extract of amla (*Emblica officinalis* Gaertn.) was investigated in human skin fibroblasts with regard to procollagen and MMP production. It was reported that the polyphenols gallic acid and elaeocarpusin present in the amla ethanolic extract enhance proliferation and collagen production in the fibroblasts. In order to minimize the formation of wrinkles, stimulation of collagen synthesis as well as the revival of damaged collagen fibers is crucial. The restoration may be achieved due to inhibition of MMPs, as demonstrated by the amla extract (in a concentration-dependent manner) [123]. The mechanism of this process was explained in the example of green tea tannin—epigallocatechin-3-gallate (EGCG)—which modulates the expression and production of matrix metalloproteinases via AP-1 and NF-κB activation [124]. Amla extract contains large amounts of tannins (emblicanin, pedunculagin, and punigluconin), which may act in a similar way to EGCG from green tea [123].

As already mentioned, many environmental factors influence the skin condition. The integrity of the skin is typically disturbed by enhanced production of matrix metalloproteinases induced by ultraviolet (UV) radiation [125]. According to Pluemsamran *et al.*, caffeic acid and ferulic acid protect human immortalized keratinocytes (HaCaT) against elevated synthesis of collagenase MMP1. The mechanism of inhibition is likely based on transcriptional and post-translational regulation of antioxidant defense [126]. A similar observation was made for pedunculagin, an ellagitannin from Mongolian Oak, which beside MMP1 inhibition stimulated procollagen production in human fibroblasts [127].

### 3.3. Attenuation of Melanin Production in Epidermis

**Figure 6:**
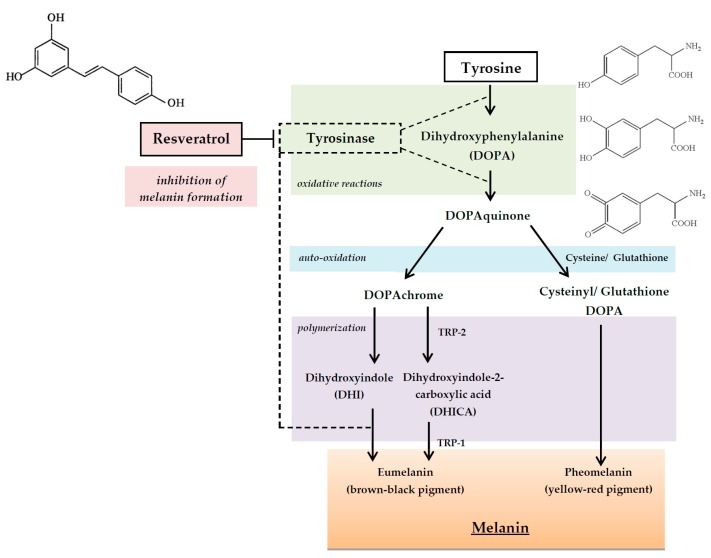
The involvement of the tyrosinase in the reaction of melanin synthesis and the inhibition of melanogenesis by the substrate analog inhibitor, *i.e.*, resveratrol. The black arrows indicate the direction of the reaction, the dash line indicates the involvement of the enzyme tyrosinase and the sign in the letter “T” shape indicates the inhibition of the tyrosinase by resveratrol.

Melanin refers to a broad spectrum of natural pigments, produced in the melanosomes—organelles localized in specialized cells called melanocytes. The basic function melanin is known to determine skin pigmentation. After its synthesis in melanocytes, melanin is transferred into the closing keratinocytes through elongated structures—dendrites. The pigment derives from an amino acid tyrosine and is formed through several oxidative reactions with the involvement of the enzyme tyrosinase (TYR) (Figure 6). The synthesis of melanin is also regulated by such enzymes as tyrosinase-related protein 1 and 2 (TRP-1, TRP-2) and cellular signaling [128,129]. Melanin has a significant role in UV protection. Melanin is critical in skin photoprotection: it is able to absorb approximately 50%–75% of UV radiation. However, the excessive production of melanin caused predominantly by UV exposure, as well as drugs, chemicals and particular disease states, may lead to dermal disorders. Hyperpigmentation is also found to be a cosmetic problem, which arises inevitably with age; hence, there is an ongoing search for new depigmenting or skin-lightening agents [130,131].

Tests performed on skin cell cultures have shown that phenolic compounds are effective in the suppression of melanin synthesis. Mitani *et al.* found that an extract of the herbal medicine Sappanwood significantly suppressed melanin production in forskolin-treated human melanoma cells (HMV-II). In the methanol extracts from Sappanwood six active compounds were identified, of which only two, brazilin and isoflavonoid-4-*O*-methylsappanol, inhibited melanogenesis [132].

The melanin inhibitors are supposed to act mostly by the suppression of tyrosinase [133,134,135]. Phenolics and flavonoids, thanks to aromatic purine rings in their composition, have similar structures to tyrosine, which are oxidized by tyrosinase, and therefore, they can act as substrate analog inhibitors against melanogenesis (Figure 6) [136,137]. Recent studies reported that resveratrol and oxyresveratrol are effective TYR inhibitors [138,139]. Unfortunately, these compounds are sensitive to photo-oxidation, which limits their use in cosmetic formulations. However, the acetylated resveratrol derivative triacetyl resveratrol is noted to be comparably effective but much more stable than resveratrol [138]. A potent suppressive impact on tyrosinase was also shown by a leaf extract from *Morus alba*, commonly used in traditional medicine. Moreover, the melanogenesis inhibitory effect occurs due to the 20 phenolic compounds determined in the extract, including eight benzofurans, 10 flavonoids (the most common being quercetin and kaempferol), one stilbenoid and one chalcone [140]. Also the extract from the small shrub *Arthrophytum scoparium* presented tyrosinase repression, predominantly due to catechol and tetrahydro-isoquinoline derivative activity. Furthermore, tyrosinase and tyrosinase-regulated gene expression were investigated in search of the potential pathway of transcriptional regulation, which suggested that inhibition of tyrosine and TRP-1 resulted from down-regulation of microphthalmia-associated transcription factor (Mitf) and melanocortin 1 receptor (Mc1R) [141]. In the research of Demirkiran *et al.*, 12 phenolic compounds determined in the *Trifolium nigrescens* Subsp. *petrisavi* were purified. Each of the identified compounds was evaluated for the inhibitory activity on mushroom tyrosinase. Among the identified phenolics three of them, which possessed the glucoside residue were found to highly inhibit the tyrosinase [142].

### 3.4. Elimination of Oxidative Stress—Protection from UV Radiation

UV radiation is considered the most frequent irritant factor for human skin cells. The UV radiation that reaches the earth surface consists of two types: (in the majority) UV-A in the wavelength range 320–400 nm and short wavelength UV-B (280–320 nm) [143]. Protection of the human skin involves endogenous antioxidant protection, melanin synthesis and antioxidant compounds—consumed or deposited on the skin. Exposure to UV results in photochemical modifications of the DNA, such as pyrimidine dimers. Although most of these changes are reversed and repaired, the accumulation of a large amount of DNA alterations may lead to permanent mutation and carcinogenesis [131,144,145].

Oxidative stress is generated during exposure to UV. It was estimated that the human skin survives 10^5^ oxidative hits daily. However, the DNA is quite stable, so the cancer induction rate is much lower than expected, considering the number of oxidative events [145]. The skin’s capacity for antioxidant self-protection rapidly decreases during aging. In order to restore the antioxidant defense properties of the skin, there is a constant need for provision of antioxidant compounds from exogenous sources, such as cosmetics or food [104]. An important example of the polyphenol, supplied from diet is epigallocatechin-3-gallate (EGCG)—the compound common in the green tea. As previously mentioned the mechanism of its action refers to the inhibition of MMPs. Moreover EGCG is very effective in improving the skin condition by the reactivation of the damaged or old cells due to the DNA protection and production of more energy in the cells [8,124].

Plant phenolic compounds are widely known for their antioxidant properties. As already mentioned, the aromatic structure of polyphenols is the significant feature in oxidative stress, namely in preventing the formation and scavenging of reactive oxygen (ROS) and nitrogen species (RNS). According to Almeida *et al.*, photoaging may be attenuated by phenolic metabolites in the presence of *Castanea sativa* polyphenols (e.g., ellagic acid). Incubation of HaCaT cells with *C. sativa* leaf extract reduced the UV-induced damage in DNA. The previous research suggests that the protective mechanism is based on direct antioxidant action (involving ^1^O_2_). Nevertheless, in this process there may occur activation of nuclear factor erythroid-related factor-2 (NRF2), a redox-sensitive transcription factor which is known to be stimulated by many phytochemical compounds [144]. The amount of UV-damaged DNA was also reduced after treatment with polyphenols (mainly anthocyanins) from berries: blackberry (*Rubus adenotrichos*), honeyberry (*Lonicera caerulea*) and bilberry (*Vaccinium myrtillus*). However, in comparison to *C. sativa* activity, after incubation with berry juices not only were the DNA mutations repaired (reduction of cyclobutane pyrimidine dimers) but also severely modified cells rapidly led to the apoptosis pathway (altered expression of caspase-3, -8, -9 genes). Apart from the cell culture, the activity of phenolics from blackberry juice was determined in a 3D skin model, while honeyberry juice activity was determined on hairless mice skin [146,147,148]. The photodamage of keratinocyte DNA caused by H_2_O_2_-induced ROS was also inhibited by oxyresveratrol and kuwanon O from *Morus australis*. The most noticeable was the reduction of pyrimidine cyclobutane dimers after treatment with *M. australis* extract [149]. The protection activity of the plant extracts from the UV-induced DNA damage was presented in the Table 3.

Apart from the DNA, UV radiation may also disturb via oxidative stress other components of the cell. The intracellular lipids are highly susceptible to the peroxidation caused by ROS formation. A study of the main *Prunella vulgaris* compound rosmarinic acid showed that UV-A-induced ROS production was suppressed and led to a decrease of lipid peroxidation [150].

UV radiation is considered the major reason for certain skin disorders such as sunburn and non-melanoma skin cancers. The application of photoprotective agents is of great importance for minimizing the harmful effect on bare skin. The effectiveness of active UV protectants is described by the sun protection factor (SPF), which is a rating of the sunburn fraction caused by UV that reaches the skin. Many natural plant polyphenols have been found to be effective as sunscreen agents. Phenolic compounds are able to absorb the radiation from the UV-B region, due to the suitable chemical structure [144,151,152]. As an example, crude extract from the medicinal plant *Schinus terebinthifolius*, as well as two extract-based formulations (gel and emulsion), showed protection of irradiated keratinocytes. The phenolic compounds identified in the extract (ethyl gallate, gallic acid and a mixture of flavonoids) were considered promising natural photoprotectants on the basis of the potent antioxidant properties and SPF assay [151].

An important aspect of photoinduced oxidative stress is the inflammatory reaction. In the literature there are many reports on attenuation of inflammatory mediators by the activity of phenolic compounds. Frequently mentioned signaling molecules are interleukin 6 (IL-6) and prostaglandin-E2 (PGE_2_). Decreased expression of the inflammatory mediators was noted after cell treatment with veratric acid [153], dihydrochalcone phloretin [154], afzelin [152], and luteolin [155].

## 4. Skin Diseases

The skin is a vital organ of the human body, which is characterized by the largest possible surface area, and it has a direct contact with the environment. Severe skin diseases such as cancer can threaten our lives. Milder disease such as acne, despite the lack of serious symptoms, often causes a lot of suffering, mainly due to the fact that the lesions are visible to other people. The development of medical knowledge and new technologies allowed scientists to significantly improve the commercially available formulations and drugs for skin treatment. Often, however, they are associated with numerous side effects, so an ideal alternative is provided by natural, bioactive components of medicinal plants such as phenolic compounds. The etiology of skin diseases may vary greatly, from environmental factors such as UV radiation and pathogens such as bacteria or fungi to genetic factors and autoimmune disorders.

**Table 3:** The protection activity of the plant extracts from the UV-induced DNA damage.

Plant (Phenolic Compounds from Extract)	Object of Study	Protection from UV-Induced DNA Damage	Results	References
*Castanea sativa* (chlorogenic acid, ellagic acid, rutin, isoquercitrin, hyperoside)	Aneuploid human immortal keratinocyte cell line (HaCaT)	Direct antioxidant action (involving ^1^O_2_); Activation of nuclear factor erythroid-related factor-2 (NRF2) gene	Protection from oxidative damage; Reduction of the UV-induced damage in DNA	[144]
Blackberry *Rubus adenotrichos* (ellagitannins, cyanidin-3-glucoside)	Normal human epidermal keratinocytes (NHEK); 3D skin model	Increased UVB-mediated poly(ADP-ribose) polymerase cleavage; activation of caspases 3, 8 and 9	Reduction of the DNA damage, including formation of cyclobutane pyrimidine dimers (CPDs) and 8-hydroxy-2′-deoxyguanosine (8-OHdG); severely modified cells were rapidly led to the apoptosis pathway	[146]
Honeyberry *Lonicera caerulea* (cyanidin-3-glucoside, caffeic acid, gallic acid)	HaCaT cell culture; Skin of albino SKH-1 hairless mice	Increased catalase activity and glutathione levels; activation of the caspases 3 and 9	Reduction of the extent of DNA breakage; decreased generation of Reactive Oxygen/ Nitrogen Species (RONS)	[147,148]
Bilberry *Vaccinium myrtillus* (cyanidin and delphinidin derivatives)	HaCaT cell culture	Activation of the caspases 3 and 9		[147]
*Morus australis* (oxyresveratrol, kuwanon O)	Human primary epidermal keratinocytes culture (HEK)	Reduction of the H_2_O_2_-induced ROS formation; p53 activation	Cellular ROS inhibition; increased cell viability; reduction of 8-OHdG and CPDs formation	[149]

### 4.1. Skin Cancers

Skin cancers are the most dangerous skin diseases. Malignancies of the skin consist of three main disorders: basal cell carcinoma, squamous cell carcinoma and malignant melanoma. The latter is less frequent than the first two kinds of cancer, but it is the most dangerous. In the early stages, the melanoma may be effectively treated by surgery, but in the severe form it often has a high mortality rate due to metastasis and resistance to chemotherapy. Epidemiological studies showed a significant increase in the incidence of melanoma in the last few years, especially for the white population [156,157,158]. High mortality of patients with melanoma has prompted many researchers to look for effective therapeutic compounds of natural origin. Plant-derived herbs and drugs were traditionally used as anti-tumor agents for many centuries and are becoming increasingly used in modern societies [159,160]. Amongst anticancer drugs currently available on the market, 60% are based on natural products and their derivatives. This has led to the increased confidence in such products as important sources for the development of effective anticancer agents [161].

**Figure 7:**
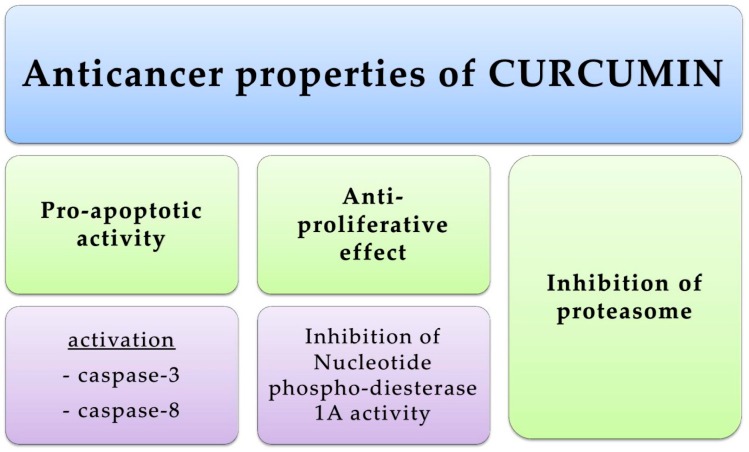
Anticancer properties of curcumin. The green fields indicate the properties of the curcumin, while violet fields indicate the particular examples of the of the curcumin action.

Promising natural compounds exhibiting anticancer properties towards skin cancers are phenolic compounds that can influence the cell cycle. One of these compounds is curcumin, which acts as a pro-apoptotic compound (Figure 7). Studies showed that this compound does not induce p53, which is important in the treatment of melanomas with a mutation of p53 which are resistant to conventional chemotherapy. Curcumin activates caspase-3 and caspase-8 but not caspase-9, and apoptosis occurs through a membrane-mediated mechanism [162,163,164]. Furthermore, curcumin has been reported to have an antiproliferative effect on a highly metastatic B16F10 murine melanoma cell line by targeting nucleotide phosphodiesterase 1A [6]. Luteolin plays a certain role in inducing cell caspase-dependant apoptosis by modulating both the extrinsic and intrinsic pathways [165]. Vitexin also shows activity in the proapoptotic process, expressed by decrease in the Bcl-2/Bax ratio and activation of caspases [166,167]. Another phenolic compound which can induce apoptosis through the activation of caspases is gallic acid (exactly caspase-3) [168].

Multidirectional antitumor activity including apoptosis induction in skin cancers was demonstrated for eugenol and its derivatives (present in aromatic plants) [169,170]. Kim *et al.* demonstrated that eugenol activates caspases, induces apoptosis and has an influence on changes in the morphology of melanoma cell nuclei [171]. Ghosh *et al.* researched the antiproliferative properties of isoeugenol [172]. They observed inhibition of the cell cycle in S phase. Moreover, in experimental animals, a decrease of metastasis and tumor invasiveness was observed. Finally, the results from cDNA array analysis indicated that the E2F transcription factors family plays a role in the eugenol-induced apoptosis in melanoma cells [172]. Eugenol not only affects melanoma development but also other skin cancers. In their study, Pal and co-werkers investigated the chemopreventive capacity of eugenol in an experimental mice skin carcinogenesis model system [173]. Histopathological analysis revealed that oral administration of eugenol could restrict the progression of carcinogenesis at the premalignant stage. It turned out that introducing eugenol treatment affected the gene expression of cancer skin cells resulting in the down-regulation of c-Myc, H-ras and Bcl2 expression up-regulation of p53, Bax along with activation of caspase-3 expression [173]. Another study by Kaur and co-workers showed that eugenol has protective activity against cutaneous chemical carcinogenesis [170]. Examples of plant extracts containing phenolic compounds with anti-apoptotic properties are the extracts from *Garcinia mangostana* L. (mangosteen) [174], propolis [175,176,177,178,179], and *Phyllanthus* [180,181,182,183,184,185] (it also has the ability to induce cell necrosis [186]).

Another important phenolic having anticancer properties is caffeic acid (3,4-dihydroxycinnamic acid). The potent favorable properties of caffeic acid on the skin cells were mentioned above with respect to the antiaging activity of polyphenols (Section 3.1 and Section 3.3). It was reported by Yang *et al.* that caffeic acid possess a strong inhibitory effect on colony formation of human skin cancer cells and epidermal growth factor (EGF)-induced neoplastic transformation of HaCaT cells dose dependently [187]. Moreover, in a solar UV-induced skin carcinogenesis mouse model topically applied to dorsal mouse skin caffeic acid greatly inhibited tumor incidence and volume. Among the mitogen-activated protein kinase (MAPK) pathway, the Ras-Raf-MEK-extracellular signal-regulated kinase 1 and 2 (ERK1/2) pathway is one of the most commonly affected in cases of human cancer [188]. Abnormalities in ERK signaling were reported in approximately 1/3 of all human cancers including skin cancers [189,190,191]. Epidemiological data indicate that solar ultraviolet (SUV) belongs to the group of the most important risk factors for development of skin cancer. SUV enhances abnormalities in ERK signaling [192,193,194]. Yang *et al.* reported the co-crystal structure of caffeic acid with ERK2 and found that caffeic acid directly inhibited SUV-induced activation of ERK1/2 and suppressed ERK signaling [181]. Caffeic acid interacted directly with ERK2, strongly binding to the ATP-binding cleft through the formation of hydrogen bonds between hydroxyl groups and amino acids Q105, D106 and M108 located at the hinge loop. Treatment of dorsal mouse skin with caffeic acid either before or after SUV exposure strongly reduced skin tumor number and size in mice [187]. Earlier studies showed that caffeic acid inhibits mouse skin cancer [195] and plays an important protective role as a screen to UVB radiation, which causes skin damage [196]. However, research carried out by Yang *et al.* showed that caffeic acid might possess anticancer activity on the molecular level [181].

Phenolic compounds, apart from the impact on the cell cycle in cancer cells, predominantly owe their anticancer properties to antioxidant activity. A number of studies point out that reactive oxygen species (ROS) and reactive nitrogen species (RNS) take part in the pathogenesis of many diseases, including cancers [197,198,199]. ROS can specifically trigger certain pathways involved in tumor proliferation, contributing to genomic instability [200]. Research suggests that providing antioxidants in the diet plays an important role in the prevention of many diseases [201,202]. An extract of the tropical plant *Remirea maritima* demonstrated selective toxicity to cancer cells when compared to normal fibroblasts cells. Chemical characterization of extracts has allowed identification of vitexin, isovitexin and luteolin [203]. These flavonoids are well known for their antioxidant properties [204,205]. Luteolin is well known for having a protective effect against H_2_O_2_-induced DNA damage. Moreover, it also acts as a protective agent against chromosomal aberrations in metastasis of malignant melanoma cells [206]. Isovitexin reduces the amount of hydrogen peroxide and inhibits both the synthesis and/or secretion of tumor necrosis factor and prostaglandin E2 (PG2) in inflammatory processes induced by lipopolysaccharide (LPS) in mouse macrophages. These findings suggested that isovitexin reduces inflammation state and carcinogenesis by the down-regulation of ROS-mediated COX-2 expression [207].

Another phenolic compounds hyperin and quercetin also possess anti-oxidative properties which makes them effective protecting agents against total reactive species, ^•^O_2_, NO^•^, and ONOO^−^. They are also known for enhancing the GSH/GSSG ratio and catalase activity in melanoma cells. In one of the most recent study it is reported that hyperin and quercetin modulate oxidative stress-induced melanogenesis not only by reducing the total level of all reactive species but also by improving the anti-oxidative ONOO− scavenging performance, the GSH/GSSG ratio and catalase activity in melanoma cells [208]. The research on Thai herbal formula conducted by Panish *et al.* revealed that it express antimelanogenic effects most probably by the stimulation of GSH and GST, which in turn improved the redox state [209].

As mentioned in Section 3.2, melanin primarily determines the color of skin and also is part of the barrier protecting the skin from UV radiation and toxic chemicals. However, excessive or abnormal accumulation of melanin in certain parts of the skin causes various pigmentation problems such as freckles, age spot, melasma, vitiligo, and skin cancer [210]. Melanogenesis inhibitors are important in the treatment of hyperpigmentation caused by excessive accumulation of melanin [211,212]. One of the active compounds that affects melanogenesis is chlorogenic acid (CGA) [213]. Research proved that it can modulate melanogenesis in B16 melanoma in two ways: CGA is a substrate for melanogenic enzymes and it inhibits enzymatic oxidation of a diphenol. CGA also reduces cell proliferation rate accelerated by 8-MOP. Other compounds possessing bioregulatory functions are tyrosine and L-DOPA, which not only induce melanogenesis but also modulate other cellular functions. To sum up, CGA has double role in melanogenesis of B16 melanoma cellsin low concentrations of CGA positively affects melanogenesis and tyrosinase activity, whereas the metabolic products of CGA may suppress melanogenesis in B16 melanoma cells. CGA is a likely substrate of melanin, but the metabolic product of CGA may suppress melanogenesis in B16 melanoma cells by inhibiting tyrosinase activity. Quercetin was reported to inhibit the formation of melanin in B16 melanoma through reduction of the intracellular activity of tyrosinase and protein expression [214,215]. Gallic acid also reduced the formation of melanin, but it showed weak inhibitory action on tyrosinase. It is suggested that gallic acid inhibits other melanin synthesis enzymes such as TRP-1 and TRP-2, MITF, TRP1, and DCT [216]. Roh *et al.* demonstrated that gallic acid and quercetin isolated from *Stewartia pseudocamellia* can inhibit melanin synthesis in melanoma cells and regulated the enzymatic activity of tyrosinase [135] Other plants rich in phenolic compounds with anti-tyrosinase activity are *Cytinus hypocistis* [134], *Magnolia grandiflora* [217] and *Zingiber officinale Rosc*. (gingerol) [218].

The mechanism of phenolic compounds’ anticancer action is based not only on their antioxidant properties, regulatory role in the cell cycle of cancer cells or influencing the melanogenesis process. Many compounds can act by inhibition of the proteasome. It is a multienzyme complex responsible for the degradation of numerous proteins involved in cell development. Inhibition of proteasome leads to the inhibition of growth and spread of cancer cells. The use of an exogenous proteasome inhibitor in the fight against cancer has created promising new therapeutic options. A wide variety of both natural and synthetic compounds, which inhibit proteasome function by binding to the active site of the catalytic subunit, is available [219,220,221]. Natural compounds that inhibit activity of the melanoma cell proteasome include syringic acid derivatives [222]. Other phenolic compounds with similar activity, a negative effect on cancer cells’ proteasome, are catechin-3-gallate and epigallocatechin gallate [223,224], gallic acid [225] apigenin, quercetin and myricetin [226], curcumin [227], and genistein [228].

The phenolic phytoalexin resveratrol is well known for its health-promoting and anticancer properties. Interesting anticancer properties are shown by pterostilbene. Being a natural dimethoxylated analog of resveratrol it exhibits much higher anticancer properies than resveratrol. Mena *et al.* showed that in cancer cells of various origin pterostilbene is an effective tool to reduce cancer cell growth, induce apoptosis and autophagosome accumulation. This compound also takes part in cancer cell death via a mechanism involving lysosomal membrane permeabilization [229]. Pterostilbene inhibits Bcl-2 expression in metastatic cells, which in turn makes them susceptible to vascular endothelium-induced cytotoxicity (a physiological defense mechanism against metastatic cell invasion) [230]. Macroautophagy is an essential conserved cellular process by which lysosomes degrade and recycle damaged organelles [231]. Its activation by polyphenols has been suggested as an possible alternative mechanism of cell death [232]. Mena *et al.* reported that pterostilbene acts in two ways, it not only induces accumulation of autophagosomes but also the conversion of cytosolic LC3-I to its lipidated membrane associated form LC3-II [229]. Moreover, pterostilbene influences lysosomal membrane permeabilization through the activation and an increase in the size of lysosomes, lysosomal membrane destabilization and intraluminal content release, induction of the release of lysosomal cathepsins and other hydrolases into the cytosol. Mena *et al.* demonstrated that the induction of cancer cell death using pterostilbene occurs mainly through lysosomal membrane permeabilization and depends on HSP70 levels [229].

**Figure 8:**
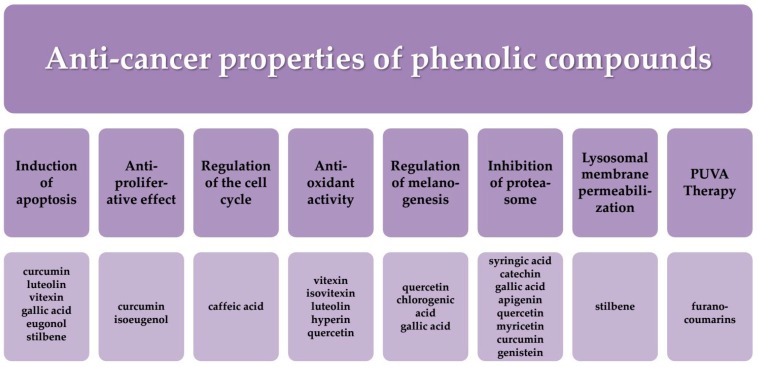
The anticancer properties of phenolic compounds.

In addition to the use of phenolic compounds in cancer therapy, as chemotherapeutic agents or diet supplements, they are also used in PUVA therapy. It is one of the therapeutic methods using light. During PUVA treatment, UVA radiation is applied with oral administration of chemical agents that sensitize the skin to the radiation. PUVA is used in the treatment of dermatological diseases such as psoriasis, vitiligo, alopecia areata acne or skin cancer [233]. In their experiments, Menichini *et al.* demonstrated that the agents which sensitize the skin to the radiation might be furanocoumarins. These compounds are found in plants belonging to the Apiaceae (Umbelliferae) family, such as celery and parsnip, Fabaceae (*Psoralea corylifolia* L.), Moraceae (fig), and Rutaceae (lemon and bergamot) [234]. Figure 8 presents the anticancer properties of phenolic compounds.

### 4.2. Psoriasis

Psoriasis is definied as a common genetic predisposition with a T-cell mediated autoimmune inflammatory skin disorder, characterized by cutaneous inflammation, increased epidermal proliferation, hyperkeratosis, angiogenesis, and abnormal keratinization. This disease is widespread all over the world and affects up to 2%–3% of the population [113,114]. Psoriasis is a disease without a lasting cure. Symptoms of psoriasis include, dry or red patches of skin covered with silvery scales and red borders. It may affect many parts of the body or all parts of the skin. However, it is commonly seen on the skin of the trunk, elbows, knees, scalps, in the finger nails and toe nails. Additional symptoms include skin lesions, cracking of skin, inflammation, itching, joints pain, increased tearing in eyes and small scaly dots on the skin especially in children. The pathophysiology of psoriasis is reckoned to associate inflammation and angiogenesis, favoring the uncontrolled outgrowth of keratinocytes. Currently, psoriasis is firmly established as strong complex genetic background along with environmental factor [235,236]. Therapeutic modalities for psoriasis comprise topical agents, phototherapies, and systemic treatments which play a role as antiproliferative agents and reduce keratinocyte proliferation [237]. Sometimes this treatment might cause irritation, phototoxicity, hypersensitivity reaction, organ toxicity, carcinogenic and broadband immunosuppression—hence natural remedies are an important alternative [236]. Various herbal constituents such as polyphenolics show immunosuppressive and anti-inflammatory activity against psoriasis [238]. This natural treatment includes a variety of natural agents, such as methanol extracts of duzhong (*Eucommia ulmoides Oliv*.), yerba mate (*Ilex paraguariensis*), linseed oil, fish oil, *Indigo naturalis* [236], and *Capsicum annum*, whose bioactive compound having antipsoriatic properties is capsaicin. Research showed a reduction of scales and redness after the application of preparations with capsaicin [239]. Other extracts in which phenolic compounds were identified and which are used in treatment of psoriasis are from the plants *Ranunculus arvensis* and *Aloe greatheadii*. Bhatti *et al.* reported that *R. arvensis* is a rich source of rutin, flavonoids and phenolics, mainly caffeic acid [240]. Compounds belonging to the classes of flavonoid and phenolics (flavonol glycosides of quercetin, kaempferol, isorhamnetin and their aglycons) were previously identified in another species of *Ranunculus* [241,242,243]. *Aloe* species have a long history in the treatment of arthritis, skin cancer, burns, eczema, psoriasis, digestive problems, high blood pressure and diabetes. Thus, natural extracts from these plants are traditionally used and commercially sold as creams, ointments and tonics. Botes *et al.* identified the active ingredients of leaf extracts from the plant. These components included in the majority phenolics from the benzoic compound group [244]. The antipsoriatic action is presented by many herbal mixtures, e.g., the combination of leaves of *Wrightia tinctoria* and *Cocos nucifera* oil rich in flavanoids, glycoflavones-iso-orientin, and phenolic acids [245,246] or the mixture of herbs used in Chinese medicine PSORI-CM01, which contains organic acids, phenolic acids, flavonoids, and terpenoids [247]. Another Chinese herbal medicine used for many years in the treatment of psoriasis is based on the plant *Scutellaria baicalensis*. The principal active ingredient is a flavonoid called baicalin. A study revealed that baicalin cream possessed anti-inflammatory action in the CHS response (2,4-dinitrofluorobenzene (DNFB)-induced contact hypersensitivity), as well as keratinocyte differentiation-modulating activity in the mouse tail test [248].

### 4.3. Rosacea

Rosacea is a chronic skin disorder whose symptoms include abnormal vascular and inflammatory conditions. Clinical manifestations might be severe and include flushing, facial erythema, inflammatory papules and pustules, telangiectasias, edema, and watery or irritated eyes [249]. Taking into account many factors, four main classes of rosacea were distinguished: erythematotelangiectatic, papulopustular, phymatous, and ocular [250]. Patients with the first subtype suffer from flushing and persistent facial erythema, sometimes accompanied by telangiectasias. The second subtype of rosacea is characterized by the presence of papules and pustules. Patients with the phymatous subtype show symptoms of skin thickening and irregular surface nodules. Patients with the ocular subtype suffer from eye irritation, which may be manifested in watery, swollen, and/or bloodshot. It has been observed that the incidence of rosacea is more common in women than in men. Some of the factors believed to trigger the onset of symptoms or exacerbate the condition include sun exposure, stress, hot and cold weather, using steroid ointments, mechanical skin damages and certain food and drinks consumption like: hot beverages, alcohol, spices and foods. The pathophysiology of rosacea is poorly understood and multifactorial. It consists of genetic, hormonal and environmental factors. In the formation of the skin lesions the most important role is attributed to immunological mechanisms and vaso-motor disturbances [249,251]. As yet, there is no effective cure for rosacea. Treatments mainly consist of relieving symptoms and inhibiting their spread, as without systematic cure the symptoms are likely to worsen. For this purpose, substances of vegetable origin are also used.

Widely used in skin care is green tea. Its main phenolic component is epigallocatechin-3-gallate (EGCG). Treatment with green tea extracts may benefit patients with a predisposition to skin conditions that present with erythema and telangiectasia. Domingo *et al.* conducted a study on the effect of a cream containing 2.5% *w*/*w* of EGCG on volunteers who for four weeks rubbed the cream on half of the face. Many data has indicated that EGCG shows an inhibitory effect on angiogenic growth and transcription factors, vascular endothelial growth factor (VEGF) and hypoxia-inducible factor-1 (HIF-1) [252]. VEGF plays a critical role in angiogenesis. Downstream of HIF-1 activation, numerous genes including VEGF are promoted, resulting in erythropoiesis, iron metabolism and angiogenesis [253,254]. EGCG as an anti-angiogenic compound may be a potential agent in the prevention of telangiectasias [252].

Research on rosacea pathogenesis suggests that major role in the process might by subscribed to the vascular changes. It was found that extract from *Chrysanthellum indicum* is rich in a unique combination of phenylpropenoic acids, flavonoids and saponosides, and, moreover, it has a well-documented effect on vascular wall permeability and mechanical resistance of capillaries. Rigopoulos *et al.* carried out research on patients with moderate rosacea. Results from an experiment with the *C. indicum* extract-based cream proved a significant decrease in the severity of erythema, overall rosacea severity compared to baseline and placebo, and investigator and patient overall efficacy assessment scores compared with placebo scores. Thus, it was concluded that *Chrysanthellum indicum* extract-based cream is a powerful and well-tolerated topical tool for the treatment of moderate rosacea [255].

### 4.4. Acne vulgaris

Acne vulgaris, although not such a serious disease as rosacea, is a very common defect which causes many physical and psychological problems. Usually it occurs during adolescence, but often it can persist throughout life and leave permanent scarring on the face. The reasons for acne development are complex, but the main factors include androgen-mediated stimulation of sebaceous gland activity, follicular hyperkeratinization, colonization of the bacterium *Propionibacterium acnes* and inflammation. Typical treatment of acne is based on local medicines, oral antibiotics, oral retinoids and oral hormonal therapies, but increasingly medicinal plants are also used. For the treatment of acne, the following properties are valued: anti-bacterial, anti-inflammatory, antioxidant, and anti-androgenic [256].

A strong antimicrobial effect against *P. acnes* strains is attributed to flavonoids isolated from *Eucalyptus maculate* extract [257], or *Terminalia arjuna* [258] and α-mangostin—the main compound in mangosteen fruit rinds [259]. Other phenolic compounds with antibacterial activity against *P. acnes* are honokiol and magnolol (isolated from *Magnolia* sp.), and gallic, chlorogenic, caffeic, ferulic, and mcinnamic acids, myricetin, quercetin, luteolin, apigenin, and thymol from wild bitter melon leaf [260]. Moreover, they have anti-inflammatory effect by reducing the secretion of IL-8 and TNF-α induced by *P. acnes*. [261]. The effect of mixtures of phenolic compounds and other drugs on *P. acnes* was also studied. Mixed formulations (kaempferol and either erythromycin or clindamycin; quercetin and either erythromycin or clindamycin) due to the synergic action caused the inhibition of antibiotic resistant *P. acnes* growth. The combination of clindamycin with kaempferol or quercetin showed a greater effect than other formulations [262].

For acne lesions, the secretion of sebum has a significant negative effect. To reduce its secretion isotretinoin and hormonal therapy is most commonly used. Yoon *et al.* reported that for this purpose epigallocatechin-3-gallate (EGCG), the main phenolic component in green tea, might be used. They demonstrated that EGCG reduced sebum excretion by modulating the AMPK–SREBP-1 signaling pathway. Moreover, the effect of EGCG extends also to reducing inflammation by suppressing the NF-κB and AP-1 pathways, induction of sebocytes cytotoxicity via apoptosis and reduction in the viability of *P. acnes*, thus having a positive influence on almost all the pathogenic features of acne. In clinical trials, this compound improved the appearance of acne lesions and was well tolerated [263].

Jumihaidokuto (JHT), a traditional Japanese medicine, has been applied for treatment of various skin disorders, including inflammatory acne. JHT contains a broad variety of polyphenols, which appeared to inhibit dermal inflammation due to their antioxidative properties via antioxidant effects. Matsumoto *et al.* reported that some flavonoid conjugates, such as genistein 7-*O*-glucuronide and liquiritigenin 7-*O*-glucuronide, were found in rat plasma where they inhibited hydrogen peroxide-dependent oxidation [264].

In the treatment of acne also anti-androgen properties of plant extracts are used. Aqueous extract from bark of the Japanese cherry tree showed a binding effect on estrogen receptor beta. In this extract sakuranetin, naringenin, genistein, genkwanin and arctigenin were investigated, from which genistein showed the strongest binding capacity [265].

### 4.5. Skin Allergies and Atopic Dermatitis

A polluted environment, processed foods, chemicals, stress—all this makes the immune system unable to cope with allergens around us and more and more people in the world suffer from skin allergies and atopic dermatitis. In the past two decades, the prevalence of allergic diseases has doubled worldwide. Changes in the eating habits are suspected to be one of the environmental factors that cause this increase and worsen allergic symptoms. When such a reason is highly likely, it is advisable to include foods or beverages with anti-allergic activities into diet, which is expected to prevent the onset of allergic and ameliorate its symptoms. In recent research, Devereux *et al.* reported that dietary antioxidant and lipid intakes during pregnancy and early childhood might lead to the lower ratio in the onset of allergic diseases [266]. In another study, Shaheen *et al.* showed that in a population-based case-control experiment in London, asthma prevalence or severity was negatively correlated with apple consumption or red wine intake, respectively, most probably due to a protective effect of flavonoids [267]. Different experiment concerned the clinical effect of certain vegetarian diets rich in flavonoids on adult patients with atopic dermatitis. It was found that, indeed, the severity of the skin diseases was alleviated, which was accompanied by the improvement in the serological parameters [268].

Compounds having such properties include also flavonoids, which possess anti-allergic activities expressed in the inhibition of histamine, IL-4, IL-13 and CD40 ligand expression by basophils and mast cells [269]. Although there is not enough clinical evidence that flavonoids prevent and mitigate allergic skin diseases, the results of various epidemiological studies, as well as the research on animal models, support this thesis [270]. The studies carried out on animal models confirmed the effectiveness mainly of quercetin and kaempferol [271,272]. During everyday-life, the skin is exposed to a broad variety of biological, chemical and physical stress factors. There is evidence these agents mediate their negative action through the ROS, which are involved in allergic and irritant contact dermatitis. In this regard, antioxidants possessed from natural products might be effective and innovative opportunity for the treatment and prevention of oxidative stress-mediated skin diseases [273]. The positive effect of *Sapium sebiferum* on allergic contact dermatitis (ACD) is due to its antioxidative properties. Seven phenolic compounds—gallic acid, ellagic acid, hyperin, isoquercitrin, astragalin, quercetin and kaempferol—were all identified in the extract from *Sapium sebiferum* leaves. Their effects on ACD were examined using a dinitrofluorobenzene (DNFB) induced mouse ACD model. The obtained results indicated that as expected, the phenolic extracts of *Sapium sebiferum* leaves inhibited the symptoms of edema induced by ACD [274]. Contact allergy involves the creation of skin lesions at the site of contact with the allergen. Common contact allergens include chemicals. Phenolic derivatives from *Phagnalon rupestre* proved to have protective activity protect against 2,4,6-trinitrochlorobenzene-induced contact hypersensitivity in mice. The following compounds were isolated from *Phagnalon rupestre*: 2-isoprenylhydroquinone-1-glucoside, 3,5-dicaffeoylquinic acid, and 3,5-dicaffeoylquinic acid methyl ester.

**Figure 9:**
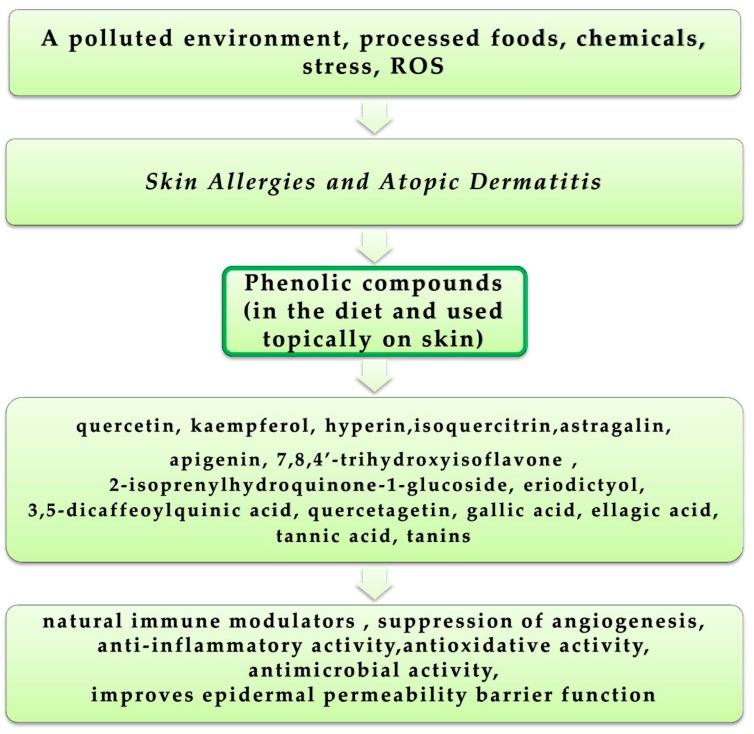
Anti-allergic properties of phenolic compounds.

Atopic dermatitis (AD) is a chronic inflammatory skin disease, and the number of patients is increasing worldwide [275]. There is a broad variety of AD clinical symptoms, which can be divided into several groups: increased levels of serum immunoglobulin E (IgE), pruritic and relapsing eczematous skin lesions characterized by epidermal thickening; defective and leaking skin barriers; and infiltration of inflammatory cells, such as lymphocytes, macrophages, eosinophils, and mast cells [275]. Agents that are perceived as playing the major roles in AD onset and development are T-helper 2 (Th2) cells producing thymus and activation-regulated chemokine (TARC), interleukin (IL)-4, IL-5, and IL-13 [276]. The main therapeutic strategy is based on treatment with steroids, but it is often associated with undesirable side effects. Hence, natural immune modulators from herbal extracts or derivatives may be useful for treating AD symptoms. Such modulators include 7,8,4’-trihydroxyisoflavone (a metabolite of soy daidzin), flavonoids from *Averrhoa carambola* L. [277,278], eriodictyol (also a flavonoid) from *Acer mono* and *Eriodictyon californicum* [279], and tannins from *Rosa multiflora* [280]. In their research on apigenin, Hou *et al.* revealed that this compound facilitates epidermal permeability barrier by stimulating epidermal differentiation, lipid synthesis and secretion, as well as cutaneous antimicrobial peptide production [281]. It is concluded, that apigenin could be an efficient tool in the prevention and treatment of skin diseases caused by dysfunctional permeability barrier associated with reduced filaggrin levels, and altered antimicrobial defenses. An example of such disorder might be atopic dermatitis [281]. Further phenolic compounds that have therapeutic properties for the treatment of AD are tannic acid and quercetin. Hung *et al.* demonstrated that these compounds act therapeutically by the suppression of angiogenesis and expression of Th2-related cytokines, including TSLP and TARC, in an atopic dermatitis (AD)-like Nc/Nga mouse model [282]. Chemokines are well known for begin the significant mediators of cell migration, for instance thymus and activation-regulated chemokine (TARC/CCL17), as well as macrophage-derived chemokine (MDC/CCL22) are commonly perceived as typical inflammatory chemokines in AD. Kang *et al.* investigated the impact of flavonoids present in unripe citruses on the levels of TARC and MDC. From the tested group of compounds, quercetagetin had the strongest inhibitory effect on the protein and mRNA expression of TARC and MDC [283]. Figure 9 presents the anti-allergic properties of phenolic compounds.

### 4.6. Dermatophytosis

Dermatophytosis is perceived as one of the most virulent diseases of humans, caused by the infection of the stratum corneum by the dermatophytic fungi *Trichophyton mentagrophytes*, *Trichophyton rubrum*, *Microsporum* spp., and *Epidermophyton* spp. [284]. Taking into account the increasing impact of these infections, the numerous hardships in their treatments (e.g., fungal resistance, side effects, and high toxicity) including its high costs, as well as the growing amount of conventional antifungals used in various industries, scientist are aiming their research at alternative natural drugs. In the light of well-known antioxidative and health-promoting properties phenolic compounds might be an ideal solution.

Such natural antifungal medicines include the extract from the green tea leaves. Park *et al.* demonstrated in *in vitro* research that green tea polyphenols (mainly catechins) can kill clinical isolates of dermatophytes [285], whereas Ikeda *et al.* proved the efficacy in clinical trials including patients with interdigital tinea pedis [286]. Apart from tannins, good antifungal activity is also shown by flavonoids. Bound flavonoids from roots of *Lantana camara* L. showed antifungal activity in studies on mice infected with *Trichophyton mentagrophytes* [287]. Complete recovery after application of the extract was observed after 16 days (drug reference after 12 days). Singh *et al.* conducted a similar study in mice using an extract of *Terminalia chebula* and found that the active compound with the greatest antifungal properties is apigenin [288]. Major biofilm-related fungal infections are caused by *Candida albicans*. They are one of the reasons for the high ratio of morbidity and mortality in hospitalized patients, especially those with immunocompromised status. Studies by Raut *et al.* revealed that phenylpropanoid compounds derived from plants are promising candidates to develop preventive line of defense against drug-resistant biofilms of *C. albicans* [289].

## 5. The Effect of Phenolic Compounds on Healing of Incised and Chronic Wounds and Burns

Healing of damaged skin is a complex and dynamic process involving both biochemical and physiological changes. In this process, there is a complex network of connections and interactions between mediators, blood cells and the extracellular matrix, which finally leads to the regeneration of the inner or outer surface of the body. Thanks to their properties, phenolic compounds may be used in the treatment of various skin damage, such as wounds and burns. Phenolic antioxidants play an important role in skin tissue repair mechanisms. In acute and chronic wounds, they may accelerate the healing process and promote proliferation of normal skin cells [290]. The antioxidative mechanism depends on inhibiting or preventing oxidation or on the conversion of hydroperoxide groups formed through the oxidative chain reaction in non-radical products [291].

### 5.1. Wounds

The process of wound healing comprises the inflammation phase, the formation of granulation tissue (reepithelization) and maturation phase. In the first phase, a clot is formed and is further infiltrated by neutrophils monocytes and thrombocytes. The next, repair phase involves the infiltration by macrophages, the formation of granulation tissue by the formation of new blood capillaries, the proliferation of fibroblasts and the transformation of the extracellular matrix. During the final stage of wound healing, granulation tissue changes into mature scar tissue as a result of resorption of exudate and strong collagen synthesis. The healing process depends on the individual characteristics of an organism, regenerative capacity and the scale of damage and can last from several weeks to several months [292,293,294].

**Figure 10:**
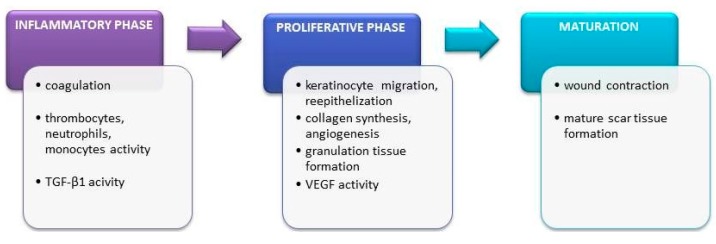
The process of wound healing.

An open wound provides a suitable environment for the growth of microorganisms. This contributes to delay or prolongation of the healing process. During the inflammatory phase, cells (neutrophils, macrophages, fibroblasts, endothelial cells) produce within the wound reactive oxygen species, thus signaling the inflammation and protecting against the invasion of microorganisms. The overproduction of reactive oxygen species can decrease the rate of wound healing and cause damage to the surrounding cells. One of the many healing mechanisms is to promote angiogenesis of the damaged tissue. Vascular endothelial growth factor (VEGF) and transforming growth factor (TGF-β1) are the effectors of angiogenesis, granulation tissue formation, collagen synthesis and deposition of extracellular matrix. Increased expression of these factors contributes to the acceleration of wound healing. Figure 10 presents the process of wound healing. Plants rich in the antioxidant phenolic compounds can effectively prevent oxidative damage of cells induced by injury in the inflammation phase, thus promoting the healing process [293,295].

The developing pharmacological market provides a variety of products supporting the healing process. Unfortunately, the side effects of these products, lack of radical treatment for some chronic wounds, the high cost of conventional medicines and microbiological resistance of strains inhabiting the wound environment lead to the increasing use of plants in medicine [112]. Phenolic compounds found in some medicinal plants in high concentrations make plants an important resource to search for new therapeutic agents. Many crude plant extracts or individual substances isolated from plants have biological activity which has been used in the treatment of skin wounds [296].

#### 5.1.1. Incised Wounds

Still a lot of research concerns the health-promoting properties of plants used in local medicine in developing countries. In Togo, a small African country situated on the Gulf of Guinea, different morphological parts of the soap berry tree (*Balanites aegyptiaca*) are traditionally used to cure many diseases and wound injuries. The health-promoting character of the plant was the background of research that proved the antioxidant and antibacterial properties of water-ethanol extract from the soap berry tree’s bark. It was revealed that this extract has antibacterial properties against *Staphylococcus aureus* and *Pseudomonas aeruginosa* strains isolated from hospitalized patients’ wounds [112]. It is also known that, e.g., sophoraflavanone G exhibits antibacterial activity, reducing the fluidity of bacterial cells, and galangin causes significant loss of potassium content in *Staphylococcus aureus* cells, which may contribute to the direct damage of the cytoplasmic membrane or indirect disturbance leading to weakening of the cell membrane and consequently to osmotic lysis [115].

On the other hand, extracts from *Clausena excavata*, *Blechnum orientale* Linn., *Anadenanthera colubrina* or *Combretum mucronatum* Schum. & Thonn, by their strong antioxidant properties, exhibit a significant effect on wound healing in rats. A reduction in the wound area, accelerated reepithelialization, stimulation cell viability of primary keratinocytes, and fibroblasts and collagen deposition compared to the control were observed [237]. Recently, it was also reported that an ethanol extract from *C. mucronatum* leaves could potentially be used to treat wounds. Although the proanthocyanidins are well known in wound healing, it has been proven for the first time that these compounds influence cell differentiation. This effect may be based on the specific interaction of procyanidin B2 with the cell cycle regulatory proteins. In the extract from *C. mucronatum* leaves apart from oligomeric proanthocyanidins and glycoside flavones (vitexin, isovitexin) the presence of flavanols (epicatechin) was also confirmed [116]. Also, the ethnotherapeutic properties of methanol extract from *Blechnum orientale* Linn., a fern widely occurring in Malaysia, were examined for the treatment of wounds in Sprague-Dawley rats. The extract was characterized by broad antibacterial activity because of the high content of tannins. When a 2% extract was applied, a significant decrease in the wound size was observed, as well as acceleration of the reepithelialization process when compared to the control [237].

Also, many herbs play an important role in wound healing. Herbs, like other plants, have a strong health-promoting potential because they promote the induction of the repair mechanisms in a natural way. The healing process with the use of bioactive plants can be physically monitored by assessing the degree of wound size reduction. A health-beneficial potential was detected in *Sphaeranthus amaranthoides* Linn., a plant growing in the wild in Asia, Africa and Australia. Studies performed to evaluate the antioxidant and health promoting activity of an extract from plant tissues growing above soil showed that the extract rich in phenolic compounds showed positive effects in the treatment of skin wounds in Wistar rats. The progress in the wound healing process was observed and measured by the speed of the contraction and wound closure and the formation of collagen. In addition, the application of the extract as a topical ointment is a promising concept due to the ease of application of ointment to large areas of wound and great exposure to the circulatory and lymphatic system, and also because of the non-invasive nature of the treatment [295].

Not only herbs and exotic plants possess health-promoting properties. This group of plants also includes industrially applicable plants. A perfect example is flax, which has been used by humans for over ten thousand years. It has been used in such branches of industry as textile or paper industry. However, in terms of health benefits special attention should be paid to the seeds, from which oil is processed. Flax fiber also has some properties useful in biomedical applications. These morphological parts of flax are rich in a variety of phenolic compounds, which are responsible for the extraordinarily valuable health-promoting benefits. Studies have investigated the impact of flax seed extract on the cut healing rate in Wistar rats. The macroscopic evaluation of the wound for 20 days showed the efficiency of the treatment, which was expressed as the decrease in the wound size and epithelialization and scar formation. Moreover, the extract from flax seeds showed a positive effect on the formation of fibrous tissue and collagen migration. Therefore, preparations based on extract from flax seed can be an effective and safe alternative to drugs used for skin regeneration [292].

#### 5.1.2. Chronic Wounds

**Figure 11:**
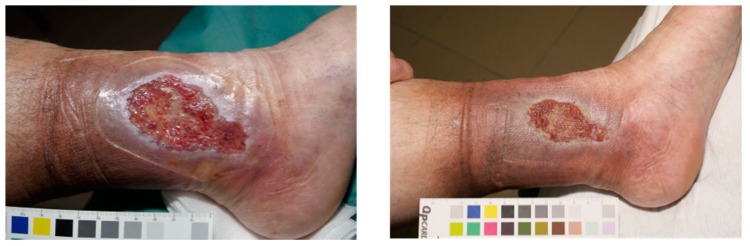
The effect of phenolic compounds derived from the flax dressing on the wound healing. Left picture presents the wound before treatment and the right presents the wound after the 12 weeks of treatment (based on the unpublished data from research of Szopa *et al.*).

Chronic wounds are those whose treatment takes longer than 8 weeks. Such wounds include venous ulcers, ischemic wounds, mainly of atherosclerosis etiology, bed sores and diabetic foot syndrome [7]. These days mainly due to inadequate nutrition habits, lifestyle diseases are a significant problem. Globally, diabetes is considered as the major one, whose incidence increases with the growing human population. Therefore, diabetic wounds as a result of the development of the disease are becoming a pressing problem. The risk of ulcers in the case of people with diabetes is around 12%–25%, and in the case of diabetic foot syndrome the risk of leg amputation is 30–40 times greater than that of ulcers with other etiology [236]. Diabetic wounds, in contrast to other wounds, heal more slowly, which in turn impedes the healing process with commercially available conventional medicines. An alternative to conventional treatment may be honey. This plant product has been used to treat wounds since ancient times, but the use of honey for treating diabetic wounds has been tested only recently. The latest clinical trials showed that the antioxidant and antibacterial activity of honey effectively accelerates the healing process of this kind of wounds. Honey has some unique features. As a natural product, in addition to the high nutritional value, it has strong antibacterial properties and is effective in preventing and combating wound infections. It is used as an agent for wound care, and it was reported to have a positive effect as an agent for the treatment of venous leg ulcers, burns and chronic leg ulcers. Due to the presence of antioxidants, including phenolic compounds, honey protects the wound surface against bacterial infections, and additionally antioxidants reduce the amount of reactive oxygen species and inflammation developing in the wound, thus helping to accelerate the healing process. Moreover, honey in many cases turns out to be a safer, faster and cheaper alternative to conventional medicines [239]. Genetically modified flax was also used to cure chronic wounds. In the Genetic Biochemistry Institute at the University of Wroclaw in Poland, genetically modified flax whose fibers were enriched in phenolics was obtained. Additionally, the seed from this flax was characterized by high content of saturated fatty acids and lignans. On this basis, linen dressings were produced, which were used in clinical trials for difficult to heal, chronic wounds. In patients suffering from chronic wounds, after application of the flax dressing significant reduction of the wound size was observed, and even complete healing after 12 weeks of treatment (Figure 11). In addition, for all the patients, a reduction in wound exudate, the level of fibrin and the presence of granulation tissue was observed. In addition, patients reported that using the dressing from genetically modified flax fiber reduced the pain associated with chronic venous ulcers [297]. Similar results were obtained using the same dressings in the treatment of a patient who suffered from bedsores. After 2.5 years of treatment, which did not bring the desired effect, we conducted a 12-weeks, four-step treatment with linen bandages. Also in this case there was a significant reduction in the size and depth of the wound, exudate and fibrin level, and pain experienced by the patient [298].

### 5.2. Burns

The treatment of burns is a complicated process. Burns can be catastrophic, causing not only physical damage but also mental and emotional disturbances [238]. Burns, due to the depth of the wound, are are divided into three grades: first degree (superficial), second degree (partial thickness) and third degree (full-thickness). The process of burns healing is complex and involves the vast number of proteins, enzymes and compounds such as: the matrix metalloproteinases (MMPs), superoxide dismutase (SOD), catalase (CAT), reduced glutathione (GSH), malondialdehyde (MDA), myeloperoxidase (MPO), vascular endothelial growth factor (VEGF), hydroxyproline, hexosamine, ascorbic acid (vitamin C), proteins found in the damaged and surrounding tissue and the cytokines interleukin-6 (IL-6) and TNF-α. In therms of severe burn wounds, they should be treated as soon as possible and with great care, because any delay or inappropriate treatment may significantly slow down the healing process or cause a severe infection. Although on the market there are several preparations for the treatment of burns, still there is a lack of ideal preparation because most of the available products have only antimicrobial activity but do not affect the wound healing process. Moreover, these products may be toxic to intact cells, and cause allergic reactions [238]. In the case of about 1% to 3% modern drugs it is suggested that they may affect both the damaged and healthy tissue. Medicinal plants rich in phenolic compounds could potentially affect the acceleration of the healing of burn wounds and protect the wound against bacterial infections [299]. Herbal products can be widely used because of their availability and extensive data about their action from traditional medicine [238]. An additional advantage of the use of herbal preparations rich in phenols in the treatment of burns is their low cost, high availability and fewer side effects [299]. A study carried out on a cream (Poly Herbal Cream) containing extract of *Malva sylvestris* L., *Rosa damascena* and *Solanum nigrum* investigated its impact on second degree burns in rats. The plant extracts were found to contain high concentrations of polyphenols and tannins. The experiments established that there was a significant improvement in the healing of burn wounds. Moreover, the wounds had fewer inflammatory cells and desired reepithelialization occurred. In addition to antioxidant activities, a cream containing the plant extract showed antibacterial activity against *Staphylococcus aureus* [238].

## 6. Conclusions

In conclusion, phenolic compounds could be potentially effective in the treatment of various skin disorders, including signs of aging, diseases and injury. Numerous studies have shown the potent biological activity of polyphenols, especially in skin cell cultures, a reconstituted skin model and rodents (mice, rats). Plant phenolics possess a significant potential to inhibit or even reverse the signs of aging, such as wrinkles or hyperpigmentation marks; hence they are promising molecules for development of new cosmetic formulations. On the other hand, particular phenolic compounds may act in a specific and effective way in order to inhibit or slow down the development of various skin-related diseases. The plant phenolics may be efficient in the treatment of both serious life-threatening dermal diseases (cancer) and minor skin problems (acne). Despite the beneficial influence of polyphenols on the skin aging effects and dermal diseases, they were also reported to be effective in healing of wounds (including chronic wounds) and burns. As regards skin disorders, therapeutic properties were shown by either a single compound or a combination of phenolics present in the plant extracts. The natural character and high effectiveness of phenolic compounds are promising features for developing novel topical formulations and dressings that might replace hitherto known remedies with limited application. Examples of phenolic compounds that possess protective and therapeutic activity in the treatment of skin disorders described in the review are summarized in Table 4.

**Table 4:** The examples of phenolic compounds that possess protective and therapeutic activity in the treatment of skin disorders. The table is based on data in the review.

Compound	Properties on Skin	References
Ellagic acid	Photoprotection, anti-allergic	[144,274]
Gallic acid	Photoprotection, maintaining skin homogeneity, anticarcinogenic, anti-acne, anti-allergic	[4,135,151,168,214,216,225,274]
Caffeic acid	Skin cells renewal, maintaining skin homogeneity, anticarcinogenic, Psoriasis treatment, anti-acne	[19,104,126,187,196,240]
Ferulic acid	Maintaining skin homogeneity, anti-acne	[19,104,126]
Quercetin	Maintaining skin homogeneity, melanogenesis inhibition, anticarcinogenic, Psoriasis treatment, anti-acne, anti-allergic, Atopic dermatitis treatment	[4,135,208,214,215,226,242,262,272,274]
Kaempferol	Melanogenesis inhibition, anti-acne, anti-allergic, Psoriasis treatment	[140,242,262]
Catechin, Epicatechin	Maintaining skin homogeneity, antibacterial, acceleration of wound healing, anticarcinogenic, Rosacea treatment, anti-acne, Dermatophytosis treatment	[4,124,223,224,252,263]
Tannins	Skin cells renewal, maintaining skin homogeneity, Atopic dermatitis treatment	[116,122,124,127,280]
Apigenin	Anticarcinogenic, anti-acne, atopic dermatitis, Dermatophytosis treatment	[226,281,288]
Luteolin	Antiinflammatory, anticarcinogenic, anti-acne	[155,165,206,261]
Vitexin	Antibacterial, acceleration of wound healing, anticarcinogenic	[9,116,166,167,217]
Isovitexin	Antibacterial, acceleration of wound healing, anticarcinogenic	[9,116,166,167,217]
Hyperin	Anticarcinogenic, anti-allergic	[208,274]
Myricetin, genistein , chlorogenic acid	Anticarcinogenic, anti-acne	[144,213,226,228,265]
Pinocembrin, pinobanksin, salicin	Skin cells renewal	[104]
Procyanidins B1, B2, caftaric acid, *trans*-resveratrol	Maintaining skin homogeneity	[4]
Brazilin, 4-*O*-methylsappanol, triacethyl resveratrol tetrahydroisoquinoline derivative	Melanogenesis inhibition	[132]
Rosmarinic acid	Photoprotection	[150]
Veratric acid, dihydrochalcone phloretin, afzelin	Antiinflammatory	[152,153,154]
Curcumin, syringic acid, pterostilbene furanocoumarins, eugenol	Anticarcinogenic	[162,163,164,222,229,234]
Capsaicin, rutin, isorhamnetin, benzoic compound, baicalin, isoorientin	Psoriasis treatment	[239,240,242,244,248]
Phenylpropenoic acids, saponosides	Rosacea treatment	[255]
Cinnamic acid, naringenin, α-mangostin, honokiol, magnolol ,thymol, 7-*O*-glucuronide, liquiritigenin 7-*O*-glucuronide, sakuranetin, genkwanin, arctigenin	Anti-acne	[260,265]
3,5-dicaffeoylquinic acid, 3,5-dicaffeoylquinic acid methyl ester, isoquercitrin, astragalin, 2-isoprenylhydroquinone-1-glucoside	Anti-allergic	[274]
Quercetagetin, 7,8,4’-trihydroxyisoflavone, eriodictyol	Atopic dermatitis treatment	[278,279,283]
Coumarin	Acceleration of wound healing	[293]
